# Supplementary material for: Serine Racemase Deletion Affects the Excitatory/Inhibitory Balance of the Hippocampal CA1 Network
Source: Int J Mol Sci. 2020 Dec 11;21(24):9447. doi: 10.3390/ijms21249447 (PMC7763099; doi:10.3390/ijms21249447)
Supplement: Supplementary file 1 [file ijms-21-09447-s001.pdf]

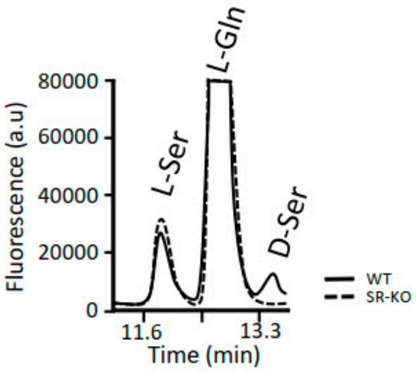

Figure S1. Representative amino acid chromatogram obtained from WT (*n* = 6) and SR-KO (*n* = 5) mice showing the peaks relative to the elution of L-serine (L-Ser), L-glutamine (L-Gln) and D-serine (D-Ser).
